# Modulation of adipose inflammation and mitochondrial pathways by a yeast-derived *β*-1,3/1,6-glucan and vitamin complex: an open-label pilot study of Lalmin® immune pro in older overweight adults

**DOI:** 10.3389/fnut.2025.1656798

**Published:** 2025-11-27

**Authors:** Jack Pritchard, Lauren Struszczak, Cealan Henry, Celeste Lugtmeijer, Joanna Bowtell, Mary O’Leary

**Affiliations:** Faculty of Health and Life Sciences, Department of Public Health and Sport Sciences, University of Exeter, Exeter, United Kingdom

**Keywords:** adipose, inflammation, beta-glucan, proteomics, aging

## Abstract

Chronic low-grade inflammation and mitochondrial dysfunction contribute to age- and obesity-related disease, yet few nutritional interventions have been shown to impact both processes. This open-label pilot study evaluated the effects of a 28-day supplementation with *Lalmin® Immune Pro—*delivering a daily dose of 250 mg yeast-derived *β*-1,3/1,6-glucan, 13.7 mg zinc, 65.0 μg selenium, and 500 IU vitamin D₂—in older, overweight adults. Proteomic profiling of subcutaneous adipose tissue was performed using tandem mass tag quantitative proteomics, with pathway-level analysis via Reactome’s CAMERA workflow. 3,172 proteins were consistently detected across all samples and used for pathway analysis. A total of 107 pathways were significantly modulated post-supplementation, including downregulation of innate (FDR = 9.3 × 10^−7^; Log2FC = −0.060) and adaptive immune pathways (FDR = 0.025; Log2FC = −0.020). Conversely, mitochondrial pathways were upregulated, including cristae formation (FDR = 6.3 × 10^−5^; Log2FC = 0.304), protein import (FDR = 1.0 × 10^−5^; Log2FC = 0.273), and respiratory electron transport (FDR = 1.4 × 10^−5^; Log2FC = 0.210). Cytokine assays of adipose explant conditioned media revealed significant reductions in the secretion of leptin (−71%), MCP-1 (−50%), IL-8 (−59%), IL-6 (−38%), and MIP-3α (−37%) post-supplementation. These findings suggest that components of Lalmin Immune Pro may exert dual immunometabolic effects, dampening inflammatory signalling while enhancing mitochondrial function in adipose tissue. Randomised controlled trials of both *Lalmin® Immune Pro and* yeast-derived *β*-1,3/1,6-glucan alone is warranted to confirm these preliminary findings and evaluate their relevance to metabolic health.

## Introduction

Obesity and advancing age remain significant public health concerns, both characterised by a state of chronic low-grade inflammation and widespread immunometabolic dysfunction. This persistent inflammatory environment plays a central role in the pathogenesis of several cardiometabolic diseases, including type 2 diabetes (T2D), cardiovascular disease (CVD), and metabolic syndrome (1, 2). As body weight increases, so does the risk for developing these diseases: individuals with overweight have approximately a twofold increased risk of cardiometabolic multimorbidity, while those with obesity experience a risk that rises exponentially—up to 14.5-fold in severe cases (3).

One of the key contributors to this inflammation-driven risk is adipose tissue. In both obesity and ageing, adipose tissue undergoes pathological expansion, leading to the development of hypertrophic and dysfunctional adipocytes. These cells release a range of pro-inflammatory mediators that initiate local inflammation and propagate systemic metabolic disturbances. The altered adipose microenvironment becomes infiltrated with immune cells such as macrophages, further amplifying inflammation (4–7). This dysfunctional adipose tissue not only contributes to systemic cytokine burden but also elevates circulating levels of free fatty acids (FFAs), contributing to insulin resistance and disrupting glucose homeostasis particularly in skeletal muscle (8, 9). In skeletal muscle, chronic exposure to inflammatory cytokines disrupts insulin signalling and mitochondrial function, driving sarcopenia and impairing regenerative capacity (10–13). Similarly, inflammation compromises endothelial function by promoting oxidative stress, reducing nitric oxide (NO) bioavailability, and impairing vascular reactivity, all of which accelerate atherosclerosis and increase the risk of CVD (14–16). Given this systemic interplay between inflamed adipose tissue and other organs, adipose tissue inflammation emerges as a critical therapeutic target for addressing cardiometabolic disease.

Lifestyle interventions such as exercise and dietary changes are proven strategies to improve metabolic health and reduce inflammation (17–20). However, the long-term adherence to these strategies can be challenging, especially among high-risk groups (21). As such, interest has grown in nutritional compounds with low barriers to adoption, such as *β*-glucans, which offer potential to modulate immune and metabolic function.

*β*-glucans are naturally occurring polysaccharides found in oats, yeast, fungi, and bacteria. They share a common backbone of β-(1,3)-linked D-glucose units, but differ in their degree and pattern of branching depending on the source. These structural variations influence their solubility, bioactivity, and immunomodulatory properties (22, 23).

Oat-derived *β*-glucans, primarily β-1,3/1,4-linked polysaccharides, are well-established for their anti-inflammatory and cardiometabolic benefits. Preclinical models show they reduce pro-inflammatory cytokines like TNF-*α* and IL-6, while human studies link them to improved glycaemic control and lower cholesterol (24–26). Mechanistically, they act through immune receptors such as Dectin-1 and toll-like receptors (TLRs), and support gut microbiota-mediated short-chain fatty acid (SCFA) production—particularly butyrate—which fosters immune regulation via Treg cell induction (27–30).

In contrast, yeast-derived *β*-glucans, which are primarily *β*-(1, 3) backbones with *β*-(1, 6) branches, are best known for enhancing immune responses during acute infections. Supplementation with yeast β-glucans has been shown to reduce the incidence and severity of upper respiratory tract infections (URTIs) in both elderly and athletic populations (31–33). These effects are attributed to the activation of innate immune pathways involving Dectin-1 and CR3, which promote phagocytosis, oxidative burst, and cytokine release (34–36). However, the immunometabolic effects of yeast-derived *β*-glucans in the absence of a pathogenic challenge remain underexplored, despite such effects being prominent in their oat-derived counterparts.

Emerging data suggest possible benefits in such contexts. In one study involving dogs with inflammatory bowel disease, supplementation with yeast-derived *β*-1,3/1,6-glucans reduced IL-6 and increased anti-inflammatory IL-10 (37). However, this mechanism was not supported in a human study where short-term β-glucan supplementation in healthy individuals did not induce measurable changes in innate immune activity (38). Nonetheless, the literature remains sparse compared to the more established anti-inflammatory benefits observed with oat *β*-glucans in metabolic disease.

Vitamin D is a fat-soluble micronutrient critical for calcium homeostasis, skeletal muscle function, and immune regulation. It is metabolised into its active form (1,25-dihydroxyvitamin D) via hydroxylation in the liver and kidneys, and exerts its effects through the vitamin D receptor, which is expressed in many immune and metabolic tissues (39). Vitamin D deficiency is widespread and associated with increased risks of CVD, muscle weakness, and impaired immune function (40). Supplementation has been shown to reduce blood pressure in hypertensive patients, improve endothelial function, and enhance muscular strength, particularly in individuals with deficiency (41–47). Zinc, an essential trace element, plays a pivotal role in innate and adaptive immunity. Zinc deficiency leads to increased oxidative stress, impaired immune cell function, and elevated pro-inflammatory cytokine production (48–51). Supplementation with zinc has been linked to reduced inflammatory markers and improved outcomes in both infectious and non-infectious inflammatory conditions (48, 50). Selenium supports immune function through its role in antioxidant defence systems, particularly via selenoproteins that regulate redox status (52–54). Low selenium levels are associated with increased oxidative stress and heightened cardiovascular risk, while supplementation may improve endothelial function and reduce markers of inflammation (55–58).

The study described in this manuscript explores the effects of a multi-nutrient intervention (Lalmin® Immune Pro) that includes yeast-derived *β*-glucans alongside vitamin D_2_ yeast, selenium-enriched yeast, and zinc-enriched yeast. Each of these components may exert independent or synergistic effects on immune and metabolic pathways. This in turn may modulate adipose tissue inflammation and improve cardiometabolic health in a population with overweight.

We hypothesised that 28 days of supplementation with Lalmin® Immune Pro, would reduce markers of chronic inflammation in human adipose tissue. To investigate this, we conducted an open-label pilot study in 15 participants, collecting subcutaneous adipose tissue and serum before and after supplementation. Tandem-mass-tagged (TMT) proteomic analysis was performed on adipose tissue samples, and *ex-vivo* experiments were conducted on adipose tissue explants. Inflammatory markers were assessed using multiplex immunoassays in both serum and culture supernatants to evaluate the immunomodulatory potential of Lalmin Immune Pro.

## Methods

### Participants

This open-label pilot study received ethical approval from the University of Exeter’s Sport and Health Sciences Research Ethics Committee (Ref: 2202391). Twenty-two older adults (≥60 years) with a BMI > 25 kg/m^2^ were screened for eligibility. Participants were recruited via a Facebook advertisement and provided written informed consent prior to screening procedures, including the completion of the Physical Activity Readiness Questionnaire (PARQ).

Fifteen participants (5 males, 10 females; age: 66.4 ± 4.6 yr.; BMI: 36.5 ± 6.1 kg/m^2^) completed the 28-day intervention. Six individuals were excluded for medical reasons and one due to supplement use that may have interfered with the study. Participants were allowed to continue prescribed medications (Supplementary Table 1) provided they did not exceed NHS-recommended dosages or conflict with the inclusion criteria. Exclusion criteria included anticoagulant use, recent NSAID/aspirin use, allergies to supplement ingredients or procedural materials (e.g., lidocaine, shellfish), skin conditions, or underlying health conditions affecting study safety or outcomes.

### Study design and supplementation

The study comprised four visits at the University of Exeter. During the baseline visit, anthropometrics were measured and study procedures explained. Participants then began a 28-day supplementation period, during which they consumed six Lalmin® Immune Pro gummies daily, delivering a total daily dose of 250 mg yeast-derived *β*-1,3/1,6-glucan (M-Gard®), 13.7 mg zinc (from Lalmin® Zn50), 65.0 μg selenium (from Lalmin® Se2000), and 500 IU vitamin D2 (from VitaD® yeast). Yeast-derived β-1,3/1,6-glucan supplementation durations of longer than 10 days have generally proved sufficient to detect changes in URTI symptoms and markers of innate immunity (59–61). Participants were recruited and the intervention took place between October 2024 and February 2025.

Samples were collected pre-supplementation (Day 0) and post-supplementation (Day 28). Participants abstained from caffeine, alcohol, and strenuous activity for 48 h prior to sampling and completed 72-h food diaries before Day 0 and Day 28, which were replicated to control for dietary variability. Participants fasted overnight prior to each sampling visit and attended the lab at the same time for each visit +/− 1 h.

Compliance was monitored by counting returned supplements. One participant was excluded from analysis due to non-compliance.

### Blood collection

Venous blood was collected into serum-separating tubes (Becton Dickinson) and centrifuged at 4500 rpm for 15 min at 4 °C. Serum was aliquoted and stored at −80 °C until analysis.

### Adipose tissue collection

Subcutaneous adipose biopsies were obtained 4–7 cm lateral to the umbilicus under local anaesthesia (2% lidocaine; B. Braun). The skin was sterilised using Videne™ or Chlorhexidine for participants with iodine/shellfish allergies. Approximately 1 g of tissue was extracted using a 14-gauge needle attached to a 50 mL syringe via the vacuum aspiration method. Contralateral sites were used for follow-up biopsies. Biopsy sites were closed using steri-strips and waterproof dressings. Tissue samples were snap-frozen in liquid nitrogen and stored at −80 °C.

### Adipose tissue explant culture

Approximately 600 mg of fresh adipose tissue was cleaned with [Phosphate-buffered saline (PBS)] and dissected into ~50 mg explants. Explants were incubated in basal endothelial media (PromoCell) in 24-well plates (300 μL per well) at 37 °C and 5% CO₂ for 24 h. Conditioned media were snap-frozen in liquid nitrogen and stored at −80 °C for cytokine analysis.

### Proteomics adipose sample preparation

Adipose tissue samples (*N* = 29) were prepared at the University of Exeter. Each sample was homogenised for 1 min using a bead homogeniser (Speedmill Plus, Analytik Jena AG), with steel beads and 250 μL of radioimmunoprecipitation assay (RIPA) buffer (Pierce 89,900 RIPA buffer, ThermoFisher Scientific) containing protease and phosphatase inhibitors (Pierce A32961 Protease and Phosphatase Inhibitor EDTA-free mini tablet, ThermoFisher Scientific). The samples were then incubated on ice for 30 min with occasional vortexing. Following incubation, the samples were centrifuged at 8000 g for 10 min at 4 °C. The supernatant was transferred to a new tube, and the cell pellet was discarded.

Protein concentrations were determined using a BCA assay (Pierce 23,225 BCA Protein Assay Kit, ThermoFisher Scientific), as per manufacturer’s instructions. Based on the BCA assay results, appropriate protein lysate dilutions were calculated, and diluted samples were prepared for proteomic analysis. A pooled sample consisting of a 1:1:1… ratio of all individual samples was also preparedas a reference sample for correction of protein load. All samples were transported to the University of Bristol for protein identification and quantification.

### TMT labelling, high pH reversed-phase chromatography

TMT proteomic analyses were conducted via one 15-plex and one 16-plex experiment. Aliquots of each sample were digested using trypsin and labelled with Tandem Mass Tag (TMTpro™) 15/16-plex reagents according to the manufacturer’s protocol (ThermoFisher Scientific, Loughborough, LE11 5RG, United Kingdom). The labelled samples were then pooled. An aliquot of the pooled sample was desalted using a Sep-Pak cartridge, following the manufacturer’s instructions (Waters, Milford, Massachusetts, United States). The eluate from the Sep-Pak cartridge was evaporated to dryness and resuspended in buffer A (20 mM ammonium hydroxide, pH 10) for subsequent fractionation by high-pH reversed-phase chromatography using an Ultimate 3,000 liquid chromatography system (ThermoFisher Scientific).

For fractionation, the sample was loaded onto an XBridge BEH C18 Column (130 Å, 3.5 μm, 2.1 mm X 150 mm, Waters, United Kingdom) in buffer A. Peptides were eluted with a gradient of buffer B (20 mM Ammonium Hydroxide in acetonitrile, pH 10) increasing from 0 to 95% over 60 min. In total, 20 fractions were collected, evaporated to dryness and resuspended in 1% formic acid before further analysis by nano-LC MS/MS using an Orbitrap Fusion Lumos mass spectrometer (Thermo Scientific).

### Nano-LC mass spectrometry

High-pH reversed-phase fractions were further separated using an Ultimate 3,000 nano-LC system coupled with an Orbitrap Fusion Lumos mass spectrometer (Thermo Scientific). Peptides resuspended in 1% (v/v) formic acid were injected into an Acclaim PepMap C18 nano-trap column (Thermo Scientific). After washing with 0.5% (v/v) acetonitrile and 0.1% (v/v) formic acid, the peptides were resolved on a 250 mm × 75 μm Acclaim PepMap C18 reverse-phase analytical column (Thermo Scientific). This was achieved over a 150-min organic gradient, comprising of seven gradient segments (1–6% solvent B over 1 min, 6–15% B over 58 min, 15–32% B over 58 min, 32–40% B over 5 min, 40–90% B over 1 min, held at 90% B for 6 min, then reduced to 1% B over 1 min) with a flow rate of 300 nL/min. Solvent A was 0.1% formic acid, and solvent B was 80% acetonitrile in 0.1% formic acid. Peptides were ionised by nano-electrospray ionisation at 2.0 kV using a stainless-steel emitter with an internal diameter of 30 μm (Thermo Scientific) and a capillary temperature of 300 °C.

All spectra were acquired using an Orbitrap Fusion Lumos mass spectrometer controlled by Xcalibur 3.0 software (Thermo Scientific), operating in data-dependent acquisition mode with an SPS-MS3 workflow. FTMS1 spectra were collected at a resolution of 120,000, with an automatic gain control (AGC) target of 200,000 and a maximum injection time of 50 ms. Precursors were filtered with an intensity threshold of 5,000, according to charge state (to include charge states 2–7), with monoisotopic peak determination set to Peptide. Previously interrogated precursors were excluded using a dynamic window (60 s ± 10 ppm). MS2 precursors were isolated with a quadrupole isolation window of 0.7 m/z. ITMS2 spectra were collected with an AGC target of 10,000, a maximum injection time of 70 ms, and CID collision energy of 35%.

For FTMS3 analysis, the Orbitrap was operated at a resolution of 50,000, with an AGC target of 50,000 and a maximum injection time of 105 ms. Precursors were fragmented by high-energy collision dissociation (HCD) with a normalised collision energy of 60% to ensure maximal TMT reporter ion yield. Synchronous Precursor Selection (SPS) was enabled to include up to 10 MS2 fragment ions in the FTMS3 scan.

### Proteomics data analysis

Raw data files were processed and quantified using Proteome Discoverer v2.4 (Thermo Scientific) and peptides were searched against the UniProt Human database (downloaded January 2024: 82415 entries) using the SEQUEST HT algorithm. The peptide precursor mass tolerance was set to 10 ppm, and MS/MS tolerance was set to 0.6 Da. Search parameters included variable modifications, such as the oxidation of methionine (+15.995 Da), acetylation of the protein N-terminus (+42.011 Da), and methionine loss plus acetylation of the protein N-terminus (−89.03 Da). Fixed modifications included Carbamidomethylation of cysteine (+57.0214) and the addition of the TMTpro™ mass tag (+304.207) to peptide N-termini and lysine residues. Searches were conducted with full tryptic digestion, allowing a maximum of two missed cleavages. The reverse database search option was enabled, and all data were filtered to satisfy a false discovery rate (FDR) of 5%.

The data were analysed at the University of Exeter. Protein quantifications were normalised to the total peptide amount in each sample and scaled against a pooled reference sample common to both TMT experiments to allow comparison across protein levels. For each protein, data were log2-transformed and filtered to include only proteins present in all samples (*n* = 3,172 proteins). Samples from participants with incomplete time points were excluded.

Further analysis was conducted using Reactome (v 89).^1^ Differential expression analysis was performed using Reactome’s ‘Correlation Adjusted Mean Rank gene set test’ (CAMERA) algorithm, which accounts for inter-protein correlations that may skew the analysis. The CAMERA algorithm adjusts the test statistics for protein sets based on these correlations. The FDR (Benjamini–Hochberg-adjusted *p*-value < 0.05) and log_2_ fold change (log_2_FC) values were calculated by Reactome and are reported for both individual proteins and pathways. Results are reported as FDR values and average fold changes (FC) derived from CAMERA analysis. Pathway results were categorised into thematic nodes, focusing on parent pathways or highest-level child pathways (FDR < 0.05) presented in biological themes.

Protein networks were visualised using Cytoscape v3.10.0, with the stringApp v2.0.1 (142). The full STRING network was applied with a high confidence threshold of 0.7. Log-transformed values (pre- vs. post-supplementation) were used to visualise the protein expression levels within the network. Network clustering was performed using the Markov clustering algorithm in the clusterMaker2 app in Cytoscape, with an inflation value set to 4.0.

### Serum and adipose-conditioned media multiplex immunoassays

Cytokine concentrations in serum and adipose-conditioned media were measured using magnetic bead-based multiplex immunoassays (Bio-Techne, LXSAHM) following manufacturer protocols. Analytes included: IL-1β, IL-6, IL-8, IL-10, IL-15, TNF-*α*, MCP-1, MIP-1α, MIP-1β, MMP-1, MMP-3, MMP-13, Eotaxin, MIP-3α, FABP4, Leptin, Leptin R, Chemerin, GP130, Resistin, Galectin-1, Aggrecan, and PBEF.

### Serum vitamin D assay

Serum samples were collected, prepared, and subsequently transported to the Blood Sciences Academic Department at the Royal Devon University NHS Foundation Trust for analysis. Quantification of total 25-hydroxyvitamin D [25(OH)D] concentration was performed using the Cobas 801 immunoassay module, integrated within the Cobas c8000 fully automated clinical chemistry platform. Each sample was analysed in accordance with the manufacturer’s instructions (Roche Diagnostics, Cobas).

### Serum short chain fatty acid quantification

All aqueous solutions were prepared using purified water at a Milli-Q grade. Acetic acid, propionic acid, butyric acid, isobutyric acid, 2-methylbutyric acid, valeric acid, isovaleric acid, caproic acid, isocaproic acid, acetic-d_3_ (ac-d4), propionic acid-d_2_ (pro-d2), butyric-d_7_ (but-d7) and 2-isobutoxyacetic acid were purchased from Sigma-Aldrich (United Kingdom). The solutions were prepared in methanol and stored at −20 °C. For derivatization 3-Nitrophenylhydrazine hydrochloride (3-NPH), 1-Ethyl-3-(3-dimethy-laminopropyl)carbodiimide hydrochloride (EDC) and pyridine were purchased by Sigma-Aldrich (United Kingdom).

80 μL serum was diluted with 720 μL ice-cold methanol and incubated on dry ice for 15 min. The samples were centrifuged (14,800 rpm, 5 min) and supernatants were filtered using 0.45 μm PTFE filters. The filtered extracts were evaporated until dry using a Savant™ SpeedVac™ High-Capacity Concentrator (Cat. SC210A-230) and were reconstituted with 40 μL of methanol. 20 μL of reconstituted sample was mixed with 20 μL of internal standard mix (20 ppm ac-d3, pro-d2, but-d7 and 2-isobutoxyacetic acid). For derivatisation, 10 μL of 3-NPH (50 mM 3-NPH solution) and 10 μL of EDC (50 mM EDC solution) made in 7% pyridine (v/v methanol) were added to the reconstituted extracts and incubated at 37 °C for 30 min in a Standard Incubator (Model B 28, Binder, Tuttlingen, Germany). The derivatisation reaction was quenched by adding 20 μL of 0.1% formic acid. All the derivatised samples were transferred to the autosampler vials containing 150 μL inserts and run on the LC–MS/MS system. Stock solutions of each metabolite were prepared in methanol (1 mg/mL) and stored at −80 °C. Calibration standards were prepared by pooling all relevant analytes for each method at 5 different concentrations by preparing a serial dilution of a solution containing acetic acid at 30 μg/mL, propionic acid at 2.5 μg/mL and the other 6 SCFA at 1.25 μg/mL and adding the respective internal standards at 50 μg/mL. Calibration standards were run at the beginning and end of each analytical queue. The analyte: internal standard response ratio was used to create calibration curves and quantify each metabolite.

Metabolite quantification was performed using liquid chromatography–tandem mass spectrometry (LC–MS/MS) comprising of Waters Acquity UPLC system and Xevo TQ-S Cronos mass spectrometer controlled by MassLynx 4.1 software. For the detection of SCFA, the electrospray ionisation (ESI) operated in negative mode and chromatographic separations were performed with a Kinetex® 2.6 μm XB-C18 (50 × 2.1 m, Phenomenex Inc.). Eluent A (0.1% formic acid, water) and eluent B (0.1% formic acid, methanol) ran at a constant rate of 0.5 mL/min. The gradient began at 10% B and was held for 1 min before a linear increase to 23% B occurred at 4.5 min, then 50% B at 6 min following by 100% B at 7 min. This was held for 0.5 min before a linear decrease in gradient back to 10% B occurred between 7.5 and 12 min. This method was adapted from Dei Cas et al. (2020). Chromatogram peak analysis was performed by the accompanying Waters® TargetLynx ™ application manager and all further data analysis and calibration curve constructions were completed in Microsoft Excel (2019 version).

Excellent linear response range was ensured for each calibration curve with correlation coefficients (r^2^) 0.99 or higher for all calibration curves generated.

### Statistical analysis

Sample size was based on the minimum effect considered sufficient to justify progression to a full trial. Specifically, we powered the study to detect a reduction in the difference between overweight and normal-weight adipose secretome profiles by approximately 50% following 4 weeks of supplementation. This 50% difference refers to the reduction in a composite measure of adipokine concentrations in adipose tissue, based on prior internal pilot data. This approach was reviewed and approved by the ethics committee as part of the sample size justification for this open-label pilot study. Using pilot data from our laboratory and standard parameters (*α* = 0.05, power = 0.8), this yielded a required sample size of 15, calculated via a two-tailed paired samples t-test.

Non-proteomics statistical analyses were performed using SPSS version 29.0.1 (IBM Corp., Armonk, NY, United States) and GraphPad Prism version 10.0 (GraphPad Software, San Diego, CA, United States). Data normality was assessed using the Shapiro–Wilk test. Due to the small sample size and non-normal distribution of some variables, non-parametric methods were applied for all adipose tissue and serum analyses.

Comparisons between pre- and post-supplementation measures were conducted using the Wilcoxon signed-rank test. Statistical significance was defined at *p* < 0.05. Data are reported as mean ± standard deviation (SD) or median with interquartile range, as appropriate.

Simple linear regression analyses were used to assess associations between changes in adipose tissue protein expression and serum biomarker levels.

## Results

### Compliance and adverse events

The average supplementation compliance rate was 93.5 ± 6.8%. One participant’s compliance was recorded at 76.1%. Their data were excluded. No adverse events were reported during the supplementation period.

### Proteomics

Proteomic analysis of subcutaneous adipose tissue identified 5,387 proteins at quantifiable levels. Among these, 3,172 proteins were present across all samples and were involved in further analysis using Reactome. No individual proteins exhibited significant differential expression between the pre-and post-supplementation groups after applying the Banjamin-Hochberg correction to Welch’s t-test results.

Reactome’s CAMERA analysis revealed significant alterations to 107 proteomic pathways following Lalmin® Immune Pro supplementation. Of these pathways, 63 were downregulated and 44 were upregulated.

**Figure 1:**
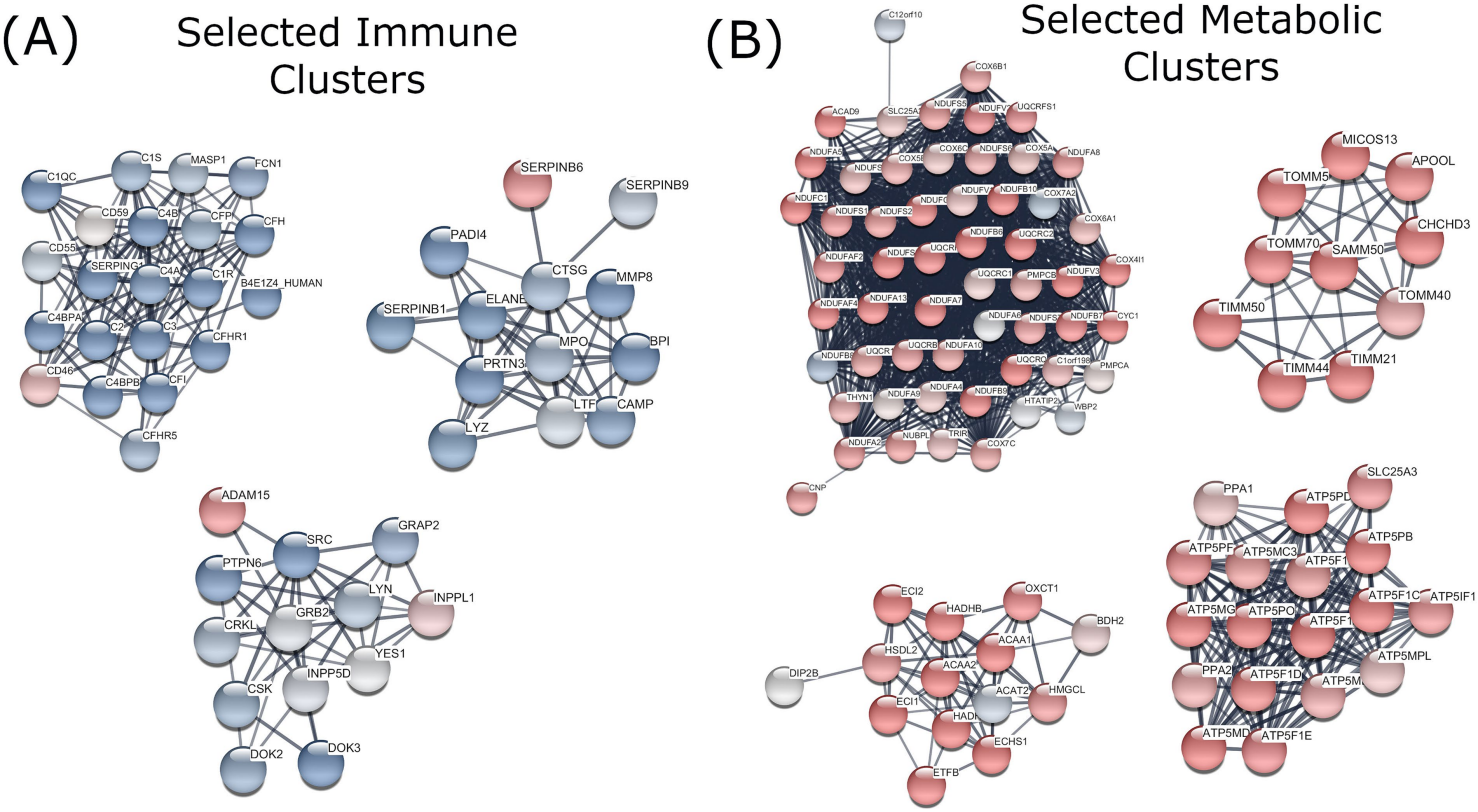
Visualisation of selected adipose tissue protein networks altered following 28 days Lalmin^®^ Immune Pro supplementation in 15 overweight/obese older adults. Log2Fold change values of protein expression from pre- to post-supplementation were mapped using Markov Clustering and manually labelled by theme. **(A)** Selected Immune Clusters **(B)** Selected Metabolic Clusters. Protein names are indicated by the official gene symbol. Blue = decreased expression, Red = increased expression.

Lalmin® Immune Pro supplementation resulted in decreased protein expression in both adaptive (FDR = 0.025, Log2FC = −0.020) and innate immune system pathways (FDR = 9.28E-07, Log2FC = −0.060). Additionally, decreased expression was observed in acute phase/haemostatic proteins (FDR = 3.72E-05, Log2FC = −0.073). Several metabolic pathways were shown to be downregulated, including the metabolism of nucleotides (FDR = 0.003, Log2FC = −0.087); plasma lipoprotein assembly, remodelling and clearance (FDR = 0.004, Log2FC = −0.146); heme scavenging from plasma (FDR = 0.182E-15, Log2FC = −0.375); and binding and uptake of ligands by Scavenger receptors (FDR = 1.79E-08, Log2FC = −0.165). A comprehensive list of protein pathways with reduced protein expression is provided in Table 1. Markov cluster visualisations support these findings (Figure 1A).

**Table 1:** Downregulated proteomic pathways in adipose tissue following 28 days Lalmin® Immune Pro supplementation in 15 overweight/obese older adults.

Pathway themes	FDR	Average Log2FC
**Adaptive Immune System**	0.025	−0.020
*Signalling by the BCR*	1.74E-05	−0.121
*Immunoregulatory Interactions between a Lymphoid and a Non-Lymphoid Cell*	1.03E-06	−0.211
**Innate Immune System**	9.82E-07	−0.060
*Complement Cascade*	7.58E-22	−0.349
*FCGR dependent Phagocytosis*	2.71E-07	−0.182
*FCERI Signalling*	1.52E-04	−0.102
*Antimicrobial Peptides*	0.003	−0.254
*Neutrophil Degranulation*	0.010	−0.030
**Hemostasias/APP**	3.72E-05	−0.073
**Metabolism of Nucleotides**	0.003	−0.087
**Plasma Lipoprotein Assembly, Remodelling, and Clearance**	0.004	−0.146
**Vesicle-mediated Transport**	-	-
*Scavenging of Heme from Plasma*	1.82E-15	−0.375
*Binding and Uptake of Ligands by Scavenger Receptors*	1.79E-08	−0.165
**LXR Regulate Gene Expression Linked to Cholesterol Transport and Efflux**	0.020	−0.194
**Regulation of IGF Transport and Uptake by IGFBPs**	0.044	−0.047
**Gamma-carboxylation, Transport, and Amino-terminal Cleavage of Proteins**	0.003	−0.348

Pathways presented are parent pathways or top-level child pathways. Differential expression was defined by FDR <0.05. Bold text = Overarching Pathway theme/Highest listed pathway, Italics = Secondary/Tertiary Pathway of the chosen theme. FDR, False Discovery Rate; FC, Fold change; BCR, B Cell Receptor; FCGR, Fcgamma Receptor; FCERI, Fc Epsilon Receptor; APP, Acute Phase Proteins; LXR, Liver X Receptors; IGF, Insulin-like Growth Factor; IGFBPs, Insulin-like Growth Factor Binding Proteins. Intensity of colour reflects extent of fold change.

Lalmin® Immune Pro supplementation resulted in the upregulation of proteins in several pathways associated with mitochondrial function (Figure 1B), including mitochondrial protein degradation (FDR = 3.03E-07, Log2FC = 0.248), cristae formation (FDR = 6.25E-05, Log2FC = 0.304), mitochondrial protein import (FDR = 1.00E-05, Log2FC = 0.273), and aerobic respiration and Respiratory electron transport (FDR = 1.40E-05, Log2FC = 0.210). Increased expression was also noted in pathways related to translation (FDR = 1.5E-04, Log2FC = 0.188) and selenoamino acid metabolism (FDR = 0.001, Log2FC = 0.221). A full list of upregulated pathways is shown in Table 2.

**Table 2:** Upregulated proteomic pathways in adipose tissue following 28 days Lalmin® Immune Pro supplementation in 15 overweight/obese older adults.

Pathway themes	FDR	Log2FC
**Aerobic Respiration and Respiratory Electron Transport**	1.40E-05	0.210
**Selenoamino Acid Metabolism**	0.001	0.221
**Translation**	1.52E-04	0.188
**Metabolism of RNA**	0.004	0.156
*rRNA Processing*	5.31E-05	0.269
*Nonsense-Mediated-Decay*	2.15E-04	0.247
**Protein Localization**	2.79E-05	0.223
*Mitochondrial Protein Import*	1.00E-05	0.273
**Cristae Formation**	6.25E-05	0.304
**Cellular Response to Starvation**	0.0312	0.198
**Fatty Acid Metabolism**	-	-
*Alpha-linolenic (omega3) and Linoleic (omega6) Acid Metabolism*	0.024	0.390
*Mitochondrial Fatty Acid Beta-Oxidation*	0.027	0.235
**Mitochondrial Protein Degradation**	3.03E-07	0.248

Pathways presented are parent pathways or top-level child pathways. Differential expression was defined by FDR <0.05. Bold text = Overarching Pathway theme/Highest listed pathway, Italics = Secondary/Tertiary Pathway of the chosen theme. FDR, False Discovery Rate; FC, Fold Change; RNA, Ribonucleic Acid; rRNA, Ribosomal Ribonucleic Acid. Intensity of colour reflects extent of fold change.

### Changes to subcutaneous adipose tissue inflammatory milieu

**Figure 2:**
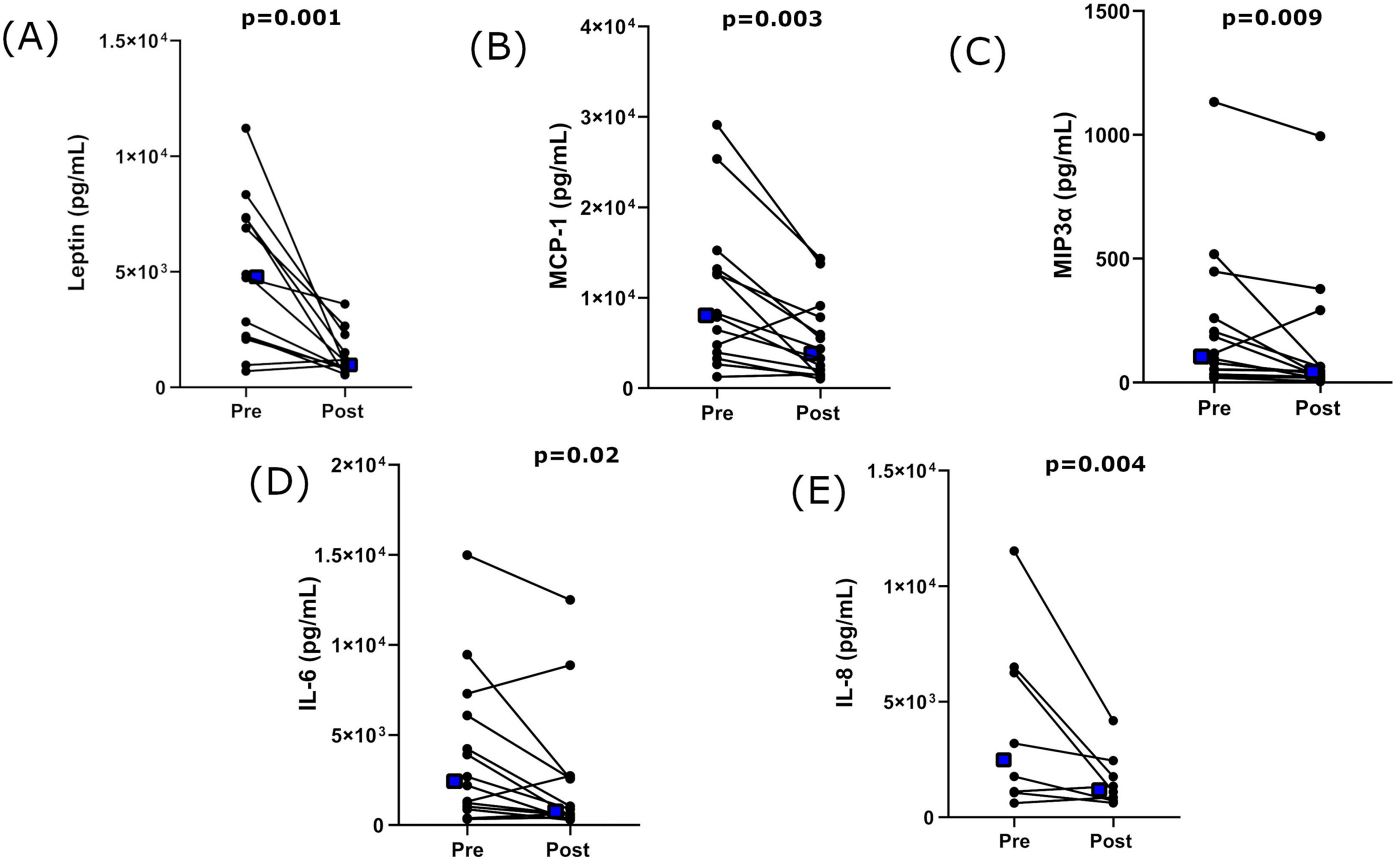
Concentrations of adipose tissue–secreted adipokines that were significantly reduced following 28 days of Lalmin^®^ Immune Pro supplementation. **(A)** Leptin, **(B)** MCP-1, **(C)** MIP3α, **(D)** IL-6, **(E)** IL-8. Data presented as individual values. Any missing values fell outside the assay range with insufficient sample available for repetition. *p* values from Wilcoxon signed rank test comparison. Blue square = mean value for each timepoint.

The concentration of adipokines secreted by subcutaneous adipose tissue was measured both pre-and post-supplementation. Significant reductions in adipose tissue secretion of leptin (post-supplementation FC = 0.3, *p* = 0.001), MCP-1 (post-supplementation FC = 0.5, *p* = 0.003), MIP-3α (post-supplementation FC = 0.56, *p* = 0.009), IL-8 (post-supplementation FC = 0.4, *p* = 0.004), and IL-6 (post-supplementation FC = 0.6, *p* = 0.020) were observed post-supplementation (Figure 2).

Results from all analytes are further presented in Supplementary Table 2 alongside means and standard deviation data.

The concentrations of serum inflammatory markers were not changed (*p* > 0.05) post-supplementation. Data are provided in Supplementary Table 2. Linear regression analyses comparing the concentrations of inflammatory markers between adipose tissue supernatants and serum were performed. Of the 22 analytes measured, only two showed significant correlations between adipose tissue supernatant and serum concentrations: FABP4 (R = 0.569, *p* < 0.001) and MMP-3 (R = 0.372, *p* = 0.001). A comprehensive overview of the correlation coefficients for each analyte is available in Supplementary Table 2.

**Figure 3:**
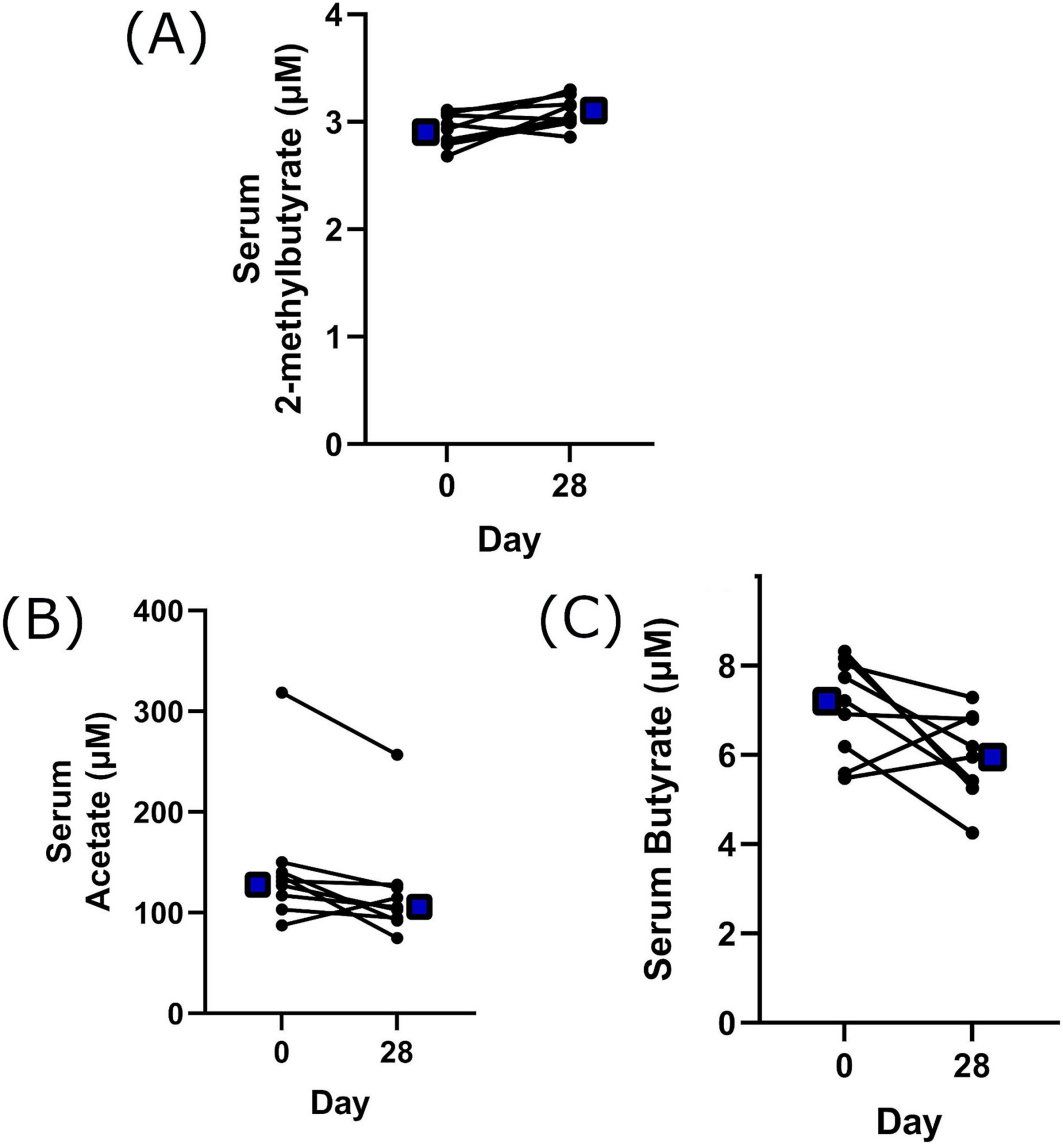
The concentration of selected serum short chain fatty acids, following 28 days Lalmin^®^ Immune Pro supplementation. Data presented as individual values. **(A)** 2-methylbutyrate, **(B)** acetate, **(C)** butyrate. Any missing values fell outside the assay range. *p* values from Wilcoxon signed rank test comparison. Blue square = mean value for each timepoint.

Acetate, propionate, isobutyrate, butyrate, 2-methylbutyrate, valerate, isocaproate, and caproate were all quantified in serum. Wilcoxon matched-pair signed-rank tests showed a significant 1.06-fold increase in 2-methylbutyrate at day 28 (*p* = 0.027). Numerical decreases in acetate and butyrate concentrations were also noted (Figure 3), but these did not reach statistical significance.

Serum vitamin D was quantified with no significant change observed following supplementation (*p* = 0.71; Supplementary Figure 1). Participants displayed a baseline mean serum vitamin D concentration of 55.7 ± 19.1 nmol/L.

## Discussion

This open-label pilot study is the first to evaluate the effect of Lalmin® Immune Pro supplementation on systemic and tissue-level inflammation, in an older, non-lean cohort. Importantly, it is also the first to assess the impact of yeast-derived *β*-glucan (1,3/1,6)—albeit in combination with micronutrients—on inflammation in the absence of pathogenic challenge. Following 4 weeks of supplementation, proteomic analysis of adipose tissue revealed significant changes in immune system proteins, particularly downregulation of those involved in innate immune responses. These changes are corroborated by our findings of reductions in adipose tissue secretion of several pro-inflammatory adipokines, such as leptin, MCP-1, MIP-3α, IL-8, and IL-6. The anti-inflammatory effects observed in adipose tissue were not evident in serum, with no reduction in serum inflammatory markers. In addition, mitochondrial proteins related to aerobic respiration and fatty acid metabolism were increased in adipose tissue.

### Immune system modulation

Our study demonstrated a significant downregulation of proteins in complement system pathways, a key component of innate immunity. This pathway is often implicated in the excessive inflammation associated with ageing and obesity—conditions typically driven by immune system dysregulation (62, 63). For example, elevated levels of serum complement C3, a central protein in the complement system, have been linked to rheumatoid arthritis, spondylarthritis, and cardiometabolic risk factors such as obesity, insulin resistance, and hypertension (64). Additional studies have further supported this connection, demonstrating an association between increased complement activity and metabolic dysfunction in obesity and ageing (65–67). Such findings indicate that Lalmin® Immune Pro supplementation could mitigate systemic inflammation and tissue damage that are prevalent in older and obese individuals at higher risk for cardiometabolic disease (64).

We observed downregulation of the Fcγ receptor-dependent phagocytosis pathway, which is crucial for macrophages to recognise and engulf antibody-coated pathogens (68, 69). This pathway is not limited to immune surveillance but also plays a critical role in maintaining immune homeostasis, however, this response can become dysregulated (70, 71). Ageing and obesity disrupt this pathway, leading to macrophage hyperactivation and chronic inflammation (72, 73). This process is further exacerbated by elevated levels of FFAs and LPS, which are commonly elevated with ageing and obesity (72, 74). This heightened macrophage activation promotes the release of pro-inflammatory cytokines (e.g., TNF-*α*, IL-6, IL-1β, MCP-1) and contributes to macrophage accumulation in adipose tissue. This results in chronic inflammation which exacerbates metabolic dysfunction and the expression of innate immune proteins (72, 75). In line with this, our data showed a reduction in adipose tissue secretion of macrophage-related adipokines, such as IL-6, IL-8, leptin, MCP-1, and MIP-3a, following supplementation with Lalmin® Immune Pro. While our results are promising, further studies are needed to validate these observations and clarify the underlying mechanisms.

We also observed a downregulation of proteins involved in neutrophil degranulation and acute-phase protein (APP) pathways, which are typically upregulated in ageing and obesity, driving chronic inflammation and metabolic dysfunction. These immune responses, usually triggered by cytokine signalling, are essential for infection and tissue repair but can cause tissue damage and sustained inflammation when dysregulated (76–80). This modulation indicates a potential for Lalmin® Immune Pro to alleviate chronic inflammation in ageing and obesity, potentially lowering cardiometabolic risk.

Adipose tissue secretion of leptin was profoundly reduced following supplementation with Lalmin® Immune Pro. Leptin is a hormone primarily secreted by adipocytes that plays a critical role in energy regulation (81, 82). It also acts as a pro-inflammatory cytokine, influencing immune cell activation and inflammatory responses (82, 83). Leptin levels are often elevated in obesity (84, 85). This elevation is linked to leptin resistance, a condition where the body becomes less responsive to leptin signalling, requiring a higher concentration to achieve its physiological effects (84). As a result, the higher levels of the pro-inflammatory cytokine during obesity contribute to chronic low-grade inflammation and can increase the risk of cardiometabolic diseases (86). The decrease in leptin secretion following supplementation indicates that Lalmin® Immune Pro may partially help restore normal leptin regulation in older overweight adults.

### Potential bioactive components driving immune modulation

Yeast *β*-glucans, although extensively studied for pathogen response modulation, have less documented effects on chronic inflammation than their oat *β*-glucan counterparts. Yeast β-glucans’ branched (1,3/1,6) structure binds strongly to immune receptors like Dectin-1 and complement receptors, enhancing immune activation in the presence of a pathogenic challenge.

Our findings show significant reduction in adipose tissue inflammation following Lalmin® Immune Pro supplementation. These dual roles likely depend on the duration and context of exposure. Indeed, exercise is well-known to have such a dual role—with acute exercise inducing a transient pro-inflammatory state (87–89) but regular exercise leads to a well-primed immune system with reduced background inflammation (90–94). The mechanisms underpinning these apparent dual roles require further exploration.

One possibility is that *β*-glucans may be metabolised into SCFAs like butyrate, which have systemic anti-inflammatory effects (29, 30). While both yeast and oat β-glucans can produce SCFAs, evidence for this process is stronger for oat β-glucans than for yeast-derived forms (27, 28, 95–97). Our data are suggestive of modest shifts in gut microbial activity following Lalmin® Immune Pro supplementation. Specifically, we observed an increase in circulating levels of 2-methylbutyrate—a branched-chain fatty acid primarily produced through microbial fermentation of amino acids (notably isoleucine). The functional significance of elevated 2-methylbutyrate in this context remains unclear (98). Notably other components of Lalmin® Immune Pro may also alter the gut microbiome and barrier function (99–102).

In contrast, no significant changes were observed in other serum SCFAs after 28 days, though we did note a trend toward reduced acetate and butyrate levels (both *p* = 0.055). While this does not support the hypothesis that yeast *β*-1,3/1,6-glucans enhance SCFA production—at least in this population and timeframe—it raises questions about temporal dynamics of circulating SCFAs and individual variability. It also calls into question the assumption that butyrate-mediated anti-inflammatory effects are a dominant mechanism of action in response to yeast β-glucan supplementation in older, overweight individuals. Longer-duration studies, with more comprehensive microbiome and metabolomic profiling, are needed to clarify whether shifts in SCFA profiles emerge over time or differ across populations.

Lalmin® Immune Pro also contains yeast-derived vitamin D, zinc, and selenium, which may synergistically modulate immunity alongside β-glucans. However, no changes in vitamin D status were observed. Zinc and selenium levels were not measured, so their contributions remain uncertain.

Vitamin D regulates inflammation and immune responses, with deficiency linked to more severe infections and chronic inflammation (41–47). Vitamin D can modulate inflammation in metabolic conditions, with a meta-analysis of 20 randomised control trials showing reductions in C-reactive protein and TNF-*α* in patients with T2D following vitamin D supplementation (103). These findings align with our observations of reduced pro-inflammatory cytokines and downregulated acute-phase protein pathways. In our cohort, four participants displayed inadequate (25–50 nmol/L) serum vitamin D concentrations, but none were deficient in vitamin D. Supplementation did not raise serum levels significantly, suggesting that the observed effects are more likely driven by other supplement constituents. However, local adipose effects cannot be dismissed (104).

Zinc plays a critical role in immune function, particularly in leukocyte recruitment and cytokine regulation. A meta-analysis of 21 RCTs reported that zinc supplementation reduced C reactive protein, TNF-α, MDA, and IL-6 (105). However, participants in these studies were zinc-deficient, and the immune effects of zinc supplementation are often most pronounced in deficient populations (106). We did not measure zinc sufficiency in our cohort, but absent deficiency the literature suggests that zinc supplementation may is unlikely to have been an important factor in our immune outcomes. Selenium’s antioxidant properties, primarily mediated by selenoproteins such as glutathione peroxidase (GPx), significantly reduce inflammation and oxidative stress (107, 108). In patients with diabetic nephropathy, selenium supplementation decreased markers like hs-CRP, MMP-2, and MDA (109). Whether these effects occur only in selenium-deficient individuals is unclear.

While vitamin D, zinc, and selenium contribute to immune modulation, their impact may only be evident for individuals with baseline deficiencies in these micronutrients. It is plausible the robust effects observed in our study stem from *β*-glucans as the principal active ingredient, but contributions by vitamin D, zinc, and selenium are possible. Further study is needed to disentangle these interactions and quantify the relative contributions of each component.

### Mitochondrial health in ageing and obesity

Mitochondrial dysfunction plays a key role in accelerating metabolic decline in both ageing and obesity (110, 111) in skeletal muscle and adipose tissue (111, 112) and other tissues. With advancing age, mitochondrial DNA integrity and mitophagy decline, leading to increased oxidative stress and reduced energy production (111, 113). Several factors contribute to this stress, including electron leakage from the electron transport chain, altered glucose and fatty acid metabolism, and inflammation. The decline in mitochondrial function further impairs the body’s ability to manage oxidative damage (114–116). In obesity, nutrient overload exacerbates mitochondrial dysfunction, further impairing energy output and increasing reliance on oxidative metabolism (110). This, in turn, leads to elevated reactive oxygen species (ROS) production, compounding oxidative stress and mitochondrial damage; such dysfunction contributes to fatigue, inflammation, and insulin resistance, ultimately promoting poor metabolic health in older obese individuals (110, 111, 117, 118).

### Upregulation of mitochondrial pathways

Lalmin® Immune Pro supplementation increased the adipose expression of proteins involved in aerobic respiration, mitochondrial function, and fuel metabolism. These pathways are critical for ATP production and modulation of ROS production (110). These changes may result from modifications in individual protein expression or changes in mitochondrial abundance.

Age impairs the function of the mitochondrial protein import system (119, 120). Lalmin® Immune Pro enhanced proteins related to protein import, which may in turn support ATP synthesis and electron transport by ensuring the availability of critical components (120). Moreover, our finding of increased proteins related to cristae formation within the inner mitochondrial membrane may increase the surface area for respiratory enzymes, optimising the arrangement of electron transport chain complexes and improving ATP production capacity (121).

Proteins related to mitochondrial protein degradation were enhanced following Lalmin® Immune Pro supplementation. Such proteins are vital in the removal of damaged mitochondrial proteins, preventing the accumulation of defective proteins and ensuring that only functional proteins are synthesised (110, 114). This coordinated response reduces ROS levels and may alleviate the inflammatory burden associated with ageing and obesity, promoting overall mitochondrial health (110).

Additionally, Lalmin® Immune Pro’s upregulation of proteins related to fatty acid metabolism and mitochondrial beta-oxidation may counteract the dysfunction of fat breakdown associated with insulin resistance during ageing and obesity (122). All of these findings require confirmation and further metabolic characterisation of tissues following Lalmin® Immune Pro supplementation is warranted, as these protein expression changes are merely a signpost towards metabolic outcomes.

These mitochondrial findings are in keeping with what is known about Lalmin’s constituents. *β*-glucans, particularly from yeast sources, enhance metabolic health by modulating fat metabolism and improving mitochondrial function in animal models (123, 124). *β*-glucan supplementation upregulates genes involved in fatty acid metabolism and lipid regulation through AMP-activated protein kinase (AMPK) signalling, promoting fat breakdown and increasing energy expenditure in mouse cell culture models (125).

Research also highlights the ability of β-glucans to reduce lipid accumulation. A study of yeast-derived β-glucan supplementation in mice pre-inoculated with gut microbiota from obese humans demonstrated upregulation of the MYC pathway, which regulates genes involved in glycolysis and lipid synthesis, leading to lower hepatic lipid levels (124). This is consistent with our findings, which show a downregulation of lipoprotein assembly and lipid-handling proteins. Further, a study of yeast-derived β-1,3/1,6 glucan supplementation in dystrophic larvae demonstrated improved locomotion and mitochondrial function (123). Supplementation significantly enhanced key measures, including basal respiration, maximum ATP production, and spare respiratory capacity (123). These effects align with the upregulated pathways we observed following Lalmin® Immune Pro supplementation in a human cohort. Vitamin D has also been suggested to influence mitochondrial function, with studies indicating that treatment with its active form results in a dose-dependent increase in mitochondrial respiration in trophoblasts from obese women (126). In skeletal muscle, *in vivo* and *in vitro* changes in vitamin D levels influence mitochondrial biogenesis and oxidative capacity (127). Vitamin D supplementation (10,000 IU vitamin D3) also improves muscle mass and strength in vitamin D-deficient older adults, likely due to enhanced mitochondrial function and ATP availability (127).

Zinc is crucial for mitochondrial function, supporting energy production through processes like glycolysis and protecting against oxidative stress (128). In animal studies, dietary zinc prevents oxidative damage to electron transport chain enzymes in malnourished rats and enhances ATP production, with zinc chelation reducing ATP generation in hepatocytes by 50% (129, 130). Zinc also modulates mitochondrial respiratory function under oxidative stress, such as ischemic conditions, improving oxidative phosphorylation and reducing ROS generation via the ERK/STAT3 pathway in isolated rat hearts (131). However, it is important to clarify the effects of zinc supplementation in humans when levels are adequate. For example, in healthy elderly individuals with adequate zinc nutrition, zinc supplementation does not effectively enhance antioxidant defence or improve redox status markers (132). Other research in this area is limited highlighting the need for more research to confirm these findings and their relevance to human mitochondrial health.

Our study revealed an upregulation of selenoamino acid metabolism, which is essential for synthesising selenoproteins, including GPx and thioredoxin reductase (TrxR). GPx plays a pivotal role in reducing hydrogen peroxide and lipid hydroperoxides, while TrxR maintains mitochondrial redox balance by reducing oxidised thioredoxin (133, 134). These processes collectively help preserve redox balance and preserve mitochondrial health. Further, the yeast derived selenium contained within Lalmin® Immune Pro can mitigate increases in plasma cholesterol and triglyceride levels and the development of atherosclerosis in hamsters fed a high-fat diet (135).

### Discrepancy between serum and tissue-specific inflammatory profiles

Despite significant reductions in inflammatory markers from adipose explants indicating local inflammation suppression, serum inflammatory markers showed little change, highlighting a disconnect between the general systemic inflammatory milieu and adipose tissue inflammation. Linear regression revealed weak correlations (below 0.6) between serum and adipose markers, with only two significant associations, suggesting systemic markers may not fully capture adipose-specific inflammation.

This aligns with existing literature showing local cytokine changes often do not reflect in plasma levels, emphasising the need to assess both tissue-specific and systemic inflammation in metabolic conditions (136). FABP4, an adipose-specific protein involved in lipid metabolism and inflammation, showed the strongest correlation, indicating its potential as a reliable serum marker of adipose inflammation (137). These findings highlight the necessity of incorporating tissue-specific analyses alongside systemic markers to understand fully the inflammatory processes in obesity and metabolic disorders.

### Study limitations and future directions

This pilot study explored the effect of Lalmin® Immune Pro on adipose and systemic inflammation, with the primary goal of assessing the utility of a larger-scale project. Accordingly, several limitations must be acknowledged, which were considered during the trial design.

The primary limitation of this study is the absence of a control group. Without a control, it is difficult to attribute the observed changes solely to Lalmin® Immune Pro, as other confounding factors may have influenced the outcomes. While our results suggest potential benefits in obese or older individuals, these findings are preliminary and should not be considered definitive. A double-blind, randomised controlled trial is required to confirm the promising effects of Lalmin® Immune Pro observed in the present study, ensuring robust control over confounding factors. Longer trials will also assess that durability of effects. None the less, the primary outcomes of this study were molecular changes in adipose tissue, in which participants baseline status acted as a control. Such outcomes are not prone to placebo effects *per se,* and we content that this is a reasonable approach in evaluating the utility of onward work.

A limitation of our study is that body weight change was not measured across the intervention. However, evidence from studies employing weight maintenance diets indicates that body weight typically exhibits minimal fluctuations over short durations. Supporting this, a four-week supplementation study using oat *β*-glucan reported no significant changes in body weight, reinforcing the notion that short-term fluctuations are negligible (138). While subtle changes in adipocyte size or tissue composition could theoretically contribute to proteomic findings, these would not be isolated from broader physiological processes in this short-term study. Current literature further suggests that substantial alterations to the proteome are typically associated with long-term changes in body weight, with little evidence to support significant short-term effects (139, 140). Nonetheless, future studies should include body weight measurements to confirm stability during the intervention period and strengthen the interpretation of proteomic findings.

It is important to note that the fold changes observed in our proteomic data were relatively modest. However, short-term molecular shifts can precede more substantial physiological adaptations over longer durations. In addition, within the proteomic analysis these fold changes represent composite effects across entire pathways. Thus, even modest overall changes may reflect meaningful modifications in pathway activity. The modest magnitude of change observed here should not necessarily be considered equivalent to the modest changes one might expect when examining an individual protein, e.g., via immunoblotting. Ultimately, proteomic analyses serve as signposts for future mechanistic work.

In addition, Lalmin® Immune Pro contains a complex combination of ingredients, including yeast-derived *β*-glucans, vitamin D, zinc and selenium. Each component is likely to influence immune function and mitochondrial function independently as well as synergistically. This makes it difficult to isolate the specific roles of each active ingredient. Therefore, further studies should focus on investigating the effects of each component to provide clearer insight into their specific contributions to inflammation and mitochondrial function. Yeast-derived *β*-glucans are, in our view, considered the most important, given the physiological effects of the other constituents are likely limited when dietary intake is sufficient. Further experimental work is required to confirm or refute this.

Our findings suggest several additional potential benefits of Lalmin® Immune Pro that warrant further investigation. Notably, the upregulation of mitochondrial pathways, particularly those related to ATP production and mitochondrial efficiency, are key factors in optimising aerobic performance (110, 120, 121). Previous studies on β-glucans demonstrate similar improvements, with increased VO₂ max and enhanced aerobic performance in athletes (141). Further studies are needed to assess if these benefits extend to older overweight populations.

Further research is needed to explore the impact of Lalmin® Immune Pro on cardio-metabolic health, particularly in older, overweight individuals and to determine if findings can be generalised to other populations. Our findings suggest this supplement may positively affect fatty acid metabolism and lipoprotein handling, potentially mitigating risks associated with metabolic syndrome, insulin resistance, and atherosclerosis. Direct measurement of other outcome measures, e.g., blood lipids, glucose handing and vascular function (e.g., flow-mediated dilatation measures) in longer-term trials are necessary to confirm these effects and assess their impact on cardiometabolic risk.

## Conclusion

This study investigated the effects of Lalmin® Immune Pro supplementation on low-grade inflammation, representing the first investigation of a multi-ingredient supplement containing yeast-derived *β*-glucan in this context, without acute infection. Over 4 weeks, we observed a significant downregulation of key immune pathways in adipose tissue proteomics, along with a reduction in the secretion of pro-inflammatory cytokines by adipose tissue after supplementation. These findings suggest that Lalmin® Immune Pro may attenuate chronic inflammation. Additionally, we identified an upregulation of mitochondrial pathways, indicating potential improvements in aerobic respiration and mitochondrial function—factors critical to age-related diseases such as obesity and metabolic dysfunction. While our study suggests the beneficial effects of Lalmin® Immune Pro for immune modulation and mitochondria function, a follow-up double-blind, randomised controlled trial is necessary to confirm these findings. Further investigating the impact of the individual components of the supplement is also recommended.

## Data Availability

Physiological and proteomics data presented in this study are deposited at: DOI: 10.24378/exe.30480473.

## Footnotes

^1^ https://reactome.org/
