# Supplementary material for: Modulation of adipose inflammation and mitochondrial pathways by a yeast-derived β-1,3/1,6-glucan and vitamin complex: an open-label pilot study of Lalmin® immune pro in older overweight adults
Source: Front Nutr. 2025 Nov 27;12:1656798. doi: 10.3389/fnut.2025.1656798 (PMC12697066; doi:10.3389/fnut.2025.1656798)
Supplement: Supplementary file 1 [file Table_1.docx]

**Supplementary Information**

**Modulation of Adipose Inflammation and Mitochondrial Pathways by a Yeast-Derived β-1,3/1,6-Glucan and Vitamin Complex: An Open-Label Pilot Study of Lalmin® Immune Pro in Older Overweight Adults.**

**Supplementary Table 1.** Medications and dietary supplements regularly taken by participants.


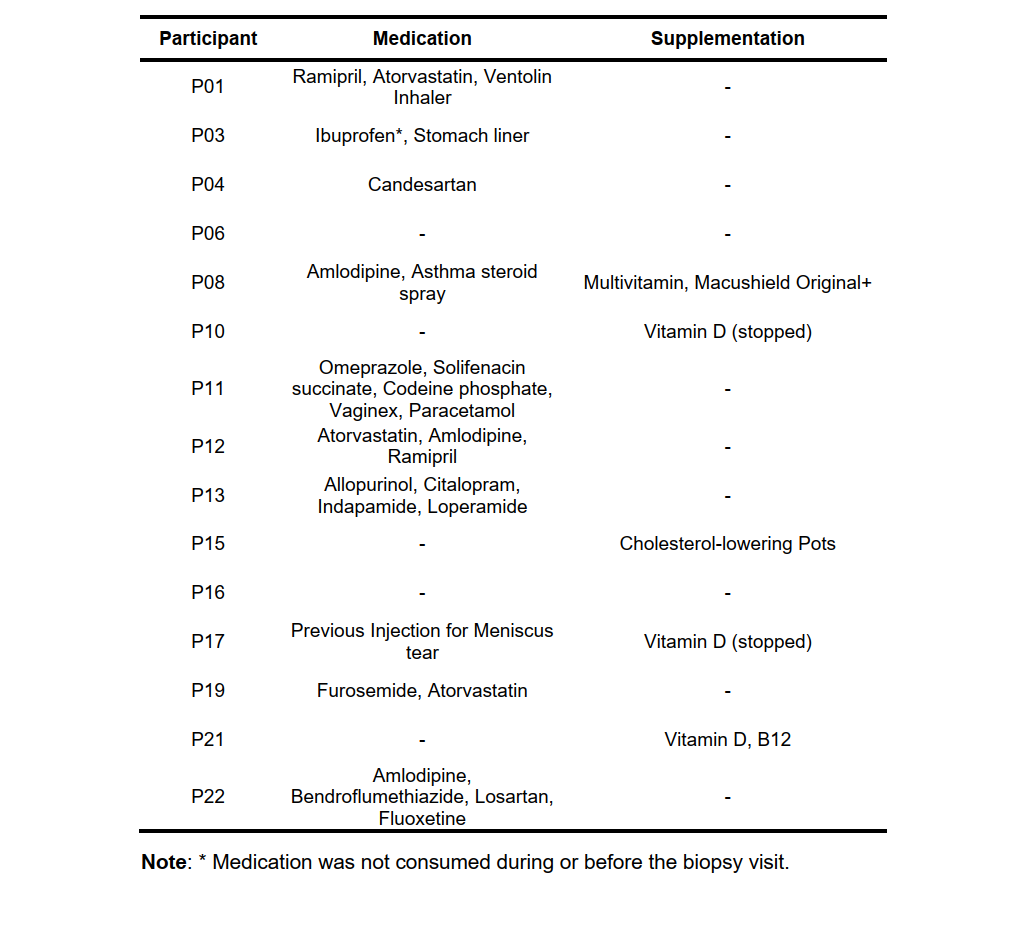


**Supplementary Table 2.** Inflammatory profile of adipose tissue supernatants and serum following Lalmin® Immune Pro supplementation, reported as (Mean ± SD pg/ml)

|  | Adipose | | | Serum | | | Adipose vs Serum | |
| --- | --- | --- | --- | --- | --- | --- | --- | --- |
| Analyte | Pre | Post | P-value | Pre | Post | P-value | R-Value | P-value |
| Aggrecan | 2916.0 ± 800.0 | 2693.2 ± 764.3 | 0.194 | 885.3 ± 254.8 | 1089.2 ± 43.6 | 0.500° | 0.130 | 0.380 |
| Chemerin | 1021.5 ± 90.1 | 1039.8 ± 142.4 | 0.626 | 14459.3 ± 4132.0 | 14578.5 ± 4203.0 | 0.910 | 0.142 | 0.063 |
| Eotaxin | 31.5 ± 14.3 | 33.2 ± 16.4 | 0.938 | 116.8 ± 54.3 | 109.3 ± 52.8 | 0.791 | 0.175 | 0.094 |
| FABP4 | 1651687.5 ± 1133921.6 | 1952763.3 ± 2337356.3 | 0.952 | 49386.8 ± 23683.7 | 56345.5 ± 39588.1 | 0.519 | 0.569 | 0.000* |
| Galectin-1 | 263026.0 ± 56960.8 | 270595.3 ± 34884.6 | 0.903 | 59731.0 ± 21209.4 | 60299.0 ± 18246.8 | 0.850 | 0.044 | 0.312 |
| GP130 | 6616.9 ± 2393.0 | 5469.4 ± 2339.7 | 0.135 | 93014.8 ± 18229.2 | 95541.2 ± 17652.5 | 0.301 | 0.000 |  |
|  |  |  |  |  |  |  |  | 0.969 |
| IL-1β | 6.4 ± 5.3 | 6.7 ± 10.2 | 0.414 | 1.2 ± 0.6 | 1.5 ± 0.5 | 0.125 | 0.011 | 0.658 |
| IL-6 | 3999.3 ± 4216.3 | 2461.2 ± 3663.1 | 0.020* | 4.2 ± 3.7 | 5.2 ± 3.6 | 0.301 | 0.043 | 0.321 |
| IL-7 | 1.7 ± 0.7 | 1.0 ± 0.6 | 0.061 | 18.3 ± 8.7 | 19.1 ± 6.7 | 0.791 | 0.008 | 0.704 |
| IL-8 | 4003.7 ± 3816.7 | 1628.2 ± 1194.1 | 0.004* | 13.5 ± 10.9 | 14.2 ± 8.7 | 0.791 | 0.058 | 0.351 |
| IL-10 | 3.07 ± 1.3 | 1.8 ± 1.0 | 0.055 | - | - | - | - | - |
| IL-15 | 4.9 ± 1.1 | 4.4 ± 2.2 | 0.233 | 1.3 ± 0.6 | 1.2 ± 0.7 | >0.999° | 0.319 | 0.089 |
| Leptin | 4744.8 ± 3253.1 | 1376.5 ± 917.9 | 0.001* | 47060.3 ± 34613.9 | 52467.2 ± 43679.1 | 0.622 | 0.152 | 0.060 |
| MCP-1 | 10473.7 ± 8365.8 | 5280.9 ± 4487.8 | 0.003* | 485.9 ± 711.7 | 464.0 ± 869.9 | 0.910 | 0.001 | 0.905 |
| MIP-1α | 104.5 ± 10.7 | 107.2 ± 27.8 | 0.414 | - | - | - | 0.531 | 0.480 |
| MIP-1β | 245.3 ± 125.0 | 201.7 ± 137.5 | 0.250 | 208.0 ± 43.9 | 212.6 ± 24.6 | 0.622 | 0.043 | 0.381 |
| MIP-3α | 229.9 ± 302.9 | 144.8 ± 268.7 | 0.009* | 113.2 ± 140.1 | 131.4 ± 139.9 | 0.102 | 0.034 | 0.389 |
| MMP-1 | 15243.9 ± 8656.6 | 14045.2 ± 9035.2 | 0.808 | 6085.4 ± 7922.1 | 4215.3 ± 3059.0 | 0.733 | 0.030 | 0.405 |
| MMP-3 | 11763.0 ± 8722.7 | 8246.0 ± 10489.1 | 0.119 | 11332.0 ± 5318.7 | 12552.4 ± 6301.4 | 0.380 | 0.372 | 0.001* |
| MMP-13 | 75.4 ± 49.4 | 81.9 ± 33.0 | 0.733 | - | - | - | - | - |
| Resistin | 7690.2 ± 8411.5 | 7228.7 ± 13866.0 | 0.583 | 16745.7 ± 7124.2 | 18042.0 ± 12120.8 | 0.622 | 0.045 | 0.309 |
| TNFα | 25.0 ± 3.2 | 25.2 ± 5.2 | >0.999 | - | - | - | 0.006 | 0.880 |

*Note. The analysis compared pre-post supplementation data (mean ±95% CI), using a Wilcoxon Sign-Rank test. P<0.05 was determined as significant (*). ° = Participant number less than 8. Missing values were due to insufficient repeats for the individual analyte.*

**
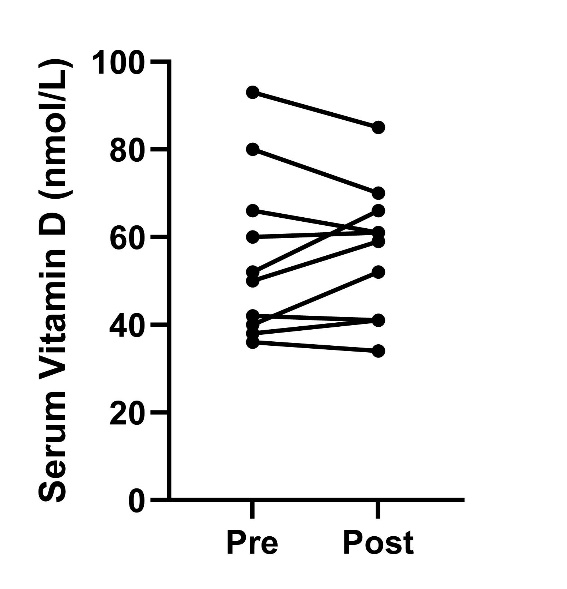
**

**Supplementary Figure 1.** **The concentration of serum vitamin D, following 28 days Lalmin® Immune Pro supplementation.** *Data presented as individual values. Any missing values are due to insufficient sample available.*
